# Linkage of genetic drivers and strain-specific germline variants confound mouse cancer genome analyses

**DOI:** 10.1038/s41467-020-18095-3

**Published:** 2020-09-08

**Authors:** Sebastian Mueller, Sebastian Lange, Katharina A. N. Collins, Stefan Krebs, Helmut Blum, Günter Schneider, Lena Rad, Dieter Saur, Roland Rad

**Affiliations:** 1 Institute of Molecular Oncology and Functional Genomics, School of Medicine, Technische Universität München, 81675 Munich, Germany; 2 Center for Translational Cancer Research (TranslaTUM), School of Medicine, Technische Universität München, 81675 Munich, Germany; 3 Department of Medicine II, Klinikum rechts der Isar, School of Medicine, Technische Universität München, 81675 Munich, Germany; 4 Laboratory for Functional Genome Analysis, Gene Center, Ludwig-Maximilians-Universität München, 81377 Munich, Germany; 5 Institute for Experimental Cancer Therapy, School of Medicine, Technische Universität München, 81675 Munich, Germany; 6 German Cancer Consortium (DKTK), German Cancer Research Center (DKFZ), 69120 Heidelberg, Germany

**Keywords:** Cancer, Cancer genomics, Cancer models

**Arising from** Niknafs et al. *Nature Communications* 10.1038/s41467-019-13100-w (2019)

Niknafs et al. describe evolutionary trajectories in pancreatic cancer using mouse models with engineered *Kras*^G12D^ and *Trp53*^R172H^ mutations (KPC model). As an additional aspect, the study reports frequent homozygous deletions at the *Nlrp1* locus, which are interpreted as a somatic driver event in pancreatic cancer. We observed that the origin of this *Nlrp1* alteration is strain-specific germline variation, having profound impact on the interpretation of its biological relevance. Beyond this specific locus, we show that strain-specific germline variation is a general confounder of genome analyses in mouse models of cancer.

In line with Niknafs et al.^1^, we also observed frequent changes at the *Nlrp1* locus in our own cohorts of KPC mice. However, *Nlrp1* changes were invariably associated with a series of unusual characteristics. First, the deletion encompasses the exact same genomic region on chromosome 11 in all affected cancers (Fig. 1a, d). These identical breakpoints in independent cancers do not reflect the typical “stepped” pattern of somatic losses at tumor suppressor loci (Fig. 1a shows such a pattern of overlaid copy number profiles). Second, the exact same deletion can also be found in other cancer entities induced in *Trp53* mutant mice, as revealed in our own studies (pancreatic cancer, osteosarcoma, lung adenocarcinoma, cutaneous squamous cell carcinoma) as well as through re-analysis of publicly available datasets (lymphomas, hepatocellular carcinomas^2–4^). Somatic acquisition of absolutely identical homozygous deletions in different cancers, models, entities, and laboratories is rather unlikely. Third, we observed *Nlrp1* locus alterations only in mouse models with engineered mutant or floxed *Trp53* alleles (*Trp53*^ENG^). More specifically, *Nlrp1* locus alterations were only detected in heterozygous *Trp53*^ENG^ tumors, but never in mice, which were crossed to *Trp53*^ENG^ homozygosity (*n* = 0/27, own cohort).

**Fig. 1:**
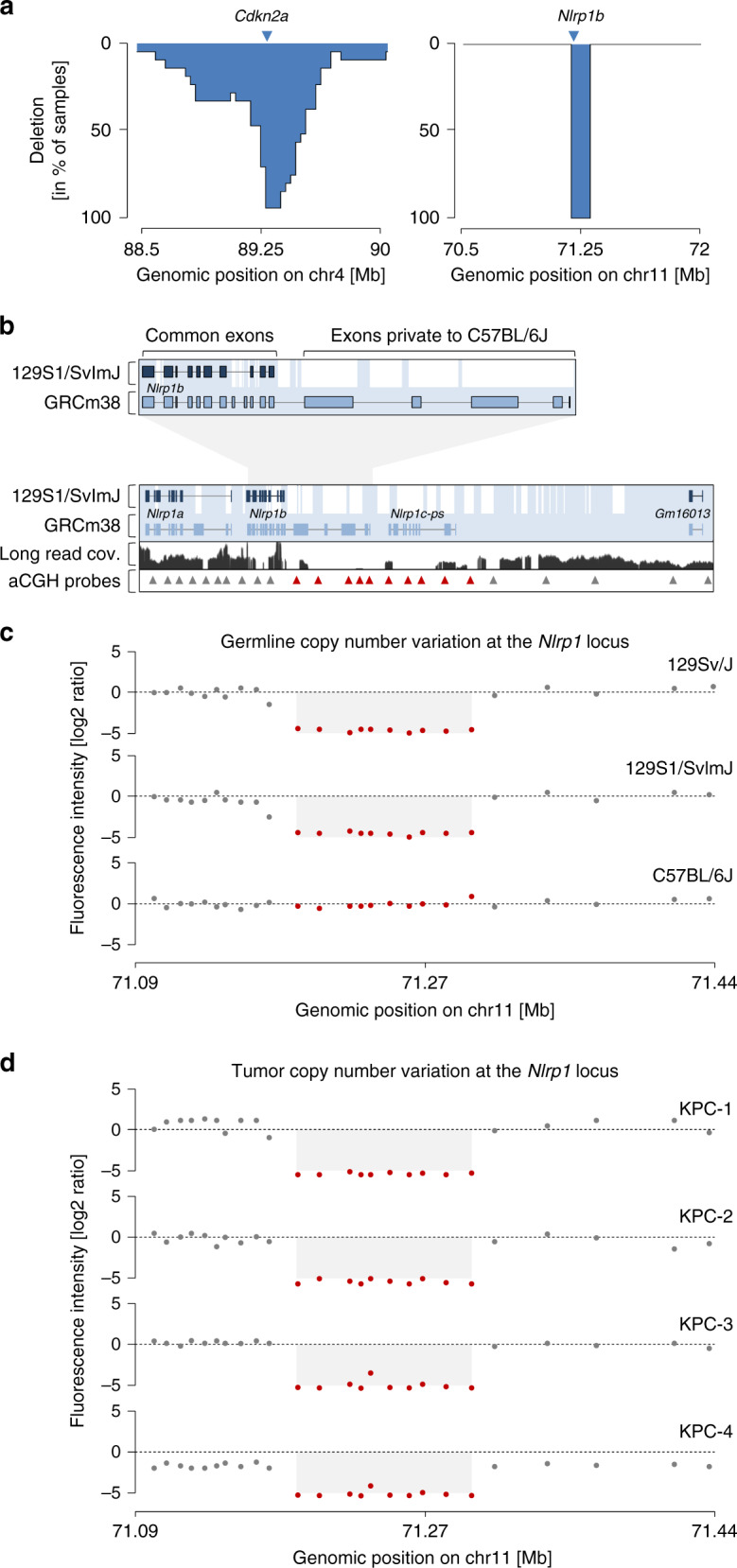
Strain-specific haplotype variation at the *Nlrp1* locus in 129S and C57BL/6J mice. **a** Overlay of homozygous somatic deletions at the “classic” tumor suppressor locus *Cdkn2a* is shown (*n* = 21 KC mice; for each tumor the homozygously deleted region is shown; data from ref. ^13^). For comparison, overlay of copy number alterations in primary pancreatic cancer cell cultures with *Nlrp1* locus deletions, as detected by aCGH (exemplary KPC mice are shown, *n* = 4; see also details of individual tumors in Fig. 1d). *Y* axis, frequency of genomic regions homozygously deleted in the cohort. **b** Strain-specific haplotype diversity at the mouse *Nlrp1* locus on chr11. Genomic alignment of the *Nlrp1*^129S1/SvImJ^ locus to *Nlrp1*^C57BL/6J^ (GRCm38 mouse reference genome). Sequence homology of C57BL/6J and 129S1/SvImJ is highlighted in light blue. Genomic regions without homology in 129S1/SvImJ are depicted in white (data adapted from ref. ^5^). Upper panel: zoom-in of *Nlrp1b* (exon/intron lengths not proportional to genomic distances). Lower panel, middle row: read coverage of the *Nlrp1*^C57BL/6J^ locus in a KPC mouse with *Trp53*^ENG^, as detected by nanopore long-read sequencing. Lower panel, bottom row: genomic position of oligonucleotide probes of the Agilent SurePrint G3 Mouse CGH 240 K array. Red arrowheads, aCGH probes located within the *Nlrp1* locus alteration described by Niknafs et al.^1^ (compare to Fig. 1c, d). **c** Germline CNV profiles at the *Nlrp1* locus in three inbred mouse strains. DNA from indicated strains was hybridized against DNA from C57BL/6J. Red dots, aCGH probes within the *Nlrp1* locus alteration (data from ref. ^6^). **d** Recurrent *Nlrp1* locus alterations in primary pancreatic cancer cell cultures with identical genomic boundaries (exemplary KPC mice are shown). The Agilent SurePrint G3 Mouse CGH 240 K array was used similar to Niknafs et al.^1^ and Fig. 1c. Red dots, aCGH probes within the *Nlrp1* locus alteration.

These seeming inconsistencies prompted us to examine the locus in detail. Humans have only one gene at this locus, *NLRP1*. In the mouse reference genome (based on strain C57BL/6J) the *Nlrp1* locus comprises three related genes: *Nlrp1a, Nlrp1b,* and *Nlrp1c-ps*. Importantly, *Trp53* and the *Nlrp1* locus are separated by only 1.5 Mb on chromosome 11, causing tight genetic linkage between both loci. The genetically engineered *Trp53* allele was generated on a 129S-related background (*Trp53*^ENG-129S^). We examined the *Nlrp1* locus in 129S genomes (*Nlrp1*^129S^) and found that parts of the C57BL/6J sequence have no genomic alignment in the 129S reference assembly^5^ (Fig. 1b). We also analyzed array comparative genomic hybridization (aCGH) data from a study examining germline copy number variation (CNV) between different mouse strains^6^. We found that genomes of 129S-related mouse strains contain homozygous deletions of the *Nlrp1* locus that were identical to *Nlrp1* locus deletions in KPC tumors (Fig. 1c, d) and all other cancer entities mentioned above. Using nanopore long-read sequencing (Fig. 1b), we confirmed the presence of the strain-specific *Nlrp1*^129S^ locus variant in the engineered *Trp53*^R172H^ mouse line^7^ used by us (and by Niknafs et al.^1^). Thus, the origin of the *Nlrp1* locus deletion is not somatic acquisition followed by selection during tumor evolution, but a pre-existing strain-specific germline variant.

After identifying that the *Trp53*^ENG-129S^ allele is genetically linked to the *Nlrp1*^129S^ locus (*Trp53*^ENG-129S^;*Nlrp1*^129S^), we interrogated the status of the second allele in the germline. This consideration is important, because we kept the mice on a mixed 129S;C57BL/6J background (similar to Niknafs et al.^1^, who used the same *Trp53*^ENG-129S^ allele on a mixed genetic background). Our analysis revealed two important findings, which explain the genesis of *Nlrp1* locus deletions in cancer: First, *Nlrp1* locus deletions in cancer were only observed in mice, whose second haplotype is of C57BL/6J origin (*Trp53*^WT^;*Nlrp1*^C57BL/6J^, Fig. 2a). Second, this C57BL/6J haplotype is lost in the tumor through copy-neutral loss of heterozygosity (CN-LOH). Mechanistically, this reflects selective pressure to lose wild-type *Trp53* during tumor progression, which almost invariably occurs in the KPC model^8^.

**Fig. 2:**
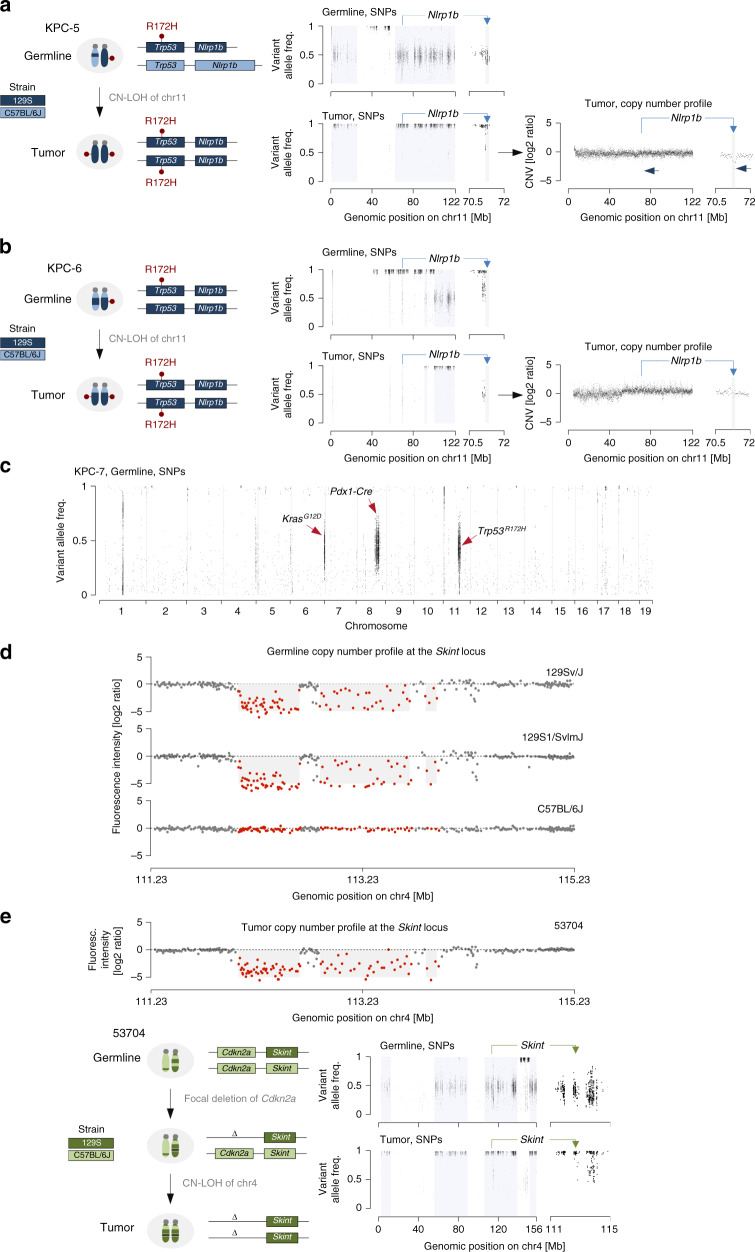
Genetic linkage of strain-specific variants to cancer genes undergoing LOH confound cancer genome analyses. **a**, **b** *Trp53*;*Nlrp1* haplotype reconstruction by WES-based SNP analysis in germline and tumors of KPC mice (**a**: KPC-5, **b**: KPC-6). Genomic regions with heterozygous SNPs contain two distinct alleles: one C57BL/6J- and one 129S-specific haplotype (SNP frequencies: ~0.5; light blue background in SNP-plots). Conversely, regions with SNP frequencies of ~1.0 are pure 129S. Regions without values perfectly match the C57BL/6J reference genome (homozygous C57BL/6J). **a** In the germline, heterozygous SNPs confirm presence of two haplotypes: (1) *Trp53*^ENG-129S^;*Nlrp1*^129S^ (engineered *Trp53*^*R172H*^ allele; strain-specific *Nlrp1*^129S^ variant) and (2) *Trp53*^WT^;*Nlrp1*^C57BL/6J^. In the tumor, the *Trp53*^WT^;*Nlrp1*^C57BL/6J^ haplotype is lost through CN-LOH (reflecting selective pressure to lose *Trp53*^WT^). Homozygosity of the *Trp53*^ENG-129S^;*Nlrp1*^129S^ haplotype in the tumor manifests as a *Nlrp1* locus deletion when compared to germline (right; CNV plot based on WES with *Nlrp1* locus zoom-in). **b** In the germline, the *Trp53*;*Nlrp1* haplotype is S129-derived on both copies of chr11. The *Nlrp1*^129S^ variant is already homozygous in the germline. CNV analyses relying on tumor/germline comparisons thus fail to detect the *Nlrp1* alteration in the tumor (right; CNV plot based on WES with *Nlrp1* locus zoom-in). **c** Germline SNP analysis of mouse KPC-7 backcrossed to C57BL/6J for fourteen generations. High SNP densities persist in genomic proximity of engineered alleles. **d** Germline CNV profiles at the *Skint* locus in three inbred mouse strains as compared to C57BL/6J. Red dots, aCGH probes within *Skint* locus alteration (data from ref. ^6^). **e** Upper panel: *Skint* locus CNV profile in primary pancreatic cancer cell culture (compare to germline *Skint* alterations in Fig. 2d). Lower panels: WES-based *Cdkn2a*;*Skint* haplotype reconstruction as in Fig. 2a, b. In the germline, heterozygous SNPs confirm presence of two haplotypes: (1) *Cdkn2a*;*Skint*^C57BL/6J^ and (2) *Cdkn2a*;*Skint*^129S^. During tumor evolution, the *Cdkn2a* locus is first somatically deleted (*Cdkn2a*^Δ^) on the chromosome carrying the *Skint*^129S^ variant (*Cdkn2a*^Δ^;*Skint*^129S^) followed by CN-LOH of the *Cdkn2a*^Δ^;*Skint*^129S^ allele, reflecting selective pressure to lose *Cdkn2a*^WT^. Homozygosity of *Skint*^129S^ in the tumor manifests as a *Skint* locus deletion when compared to germline (right; CNV plot based on WES with *Skint* locus zoom-in).

Because detection of CNVs is based on the comparison of tumor to germline, these findings explain: (1) why a single somatic event (loss of *Trp53*^WT^;*Nlrp1*^C57BL/6J^ through CN-LOH) manifests as a focal homozygous deletion (Fig. 2a), (2) why the *Nlrp1*^129S^ variant is not detected (despite being present) in tumors from mice crossed to *Trp53*^ENG^ homozygosity (*Trp53*^ENG-129S^;*Nlrp1*^129S^ already homozygous in the germline) or other more rare scenarios (Fig. 2b) and (3) why the coordinates of the *Nlrp1*^129S^ variant are identical in tumors across models and entities (see Fig. 1a, d).

Is the *Nlrp1*^129S^ variant biologically relevant and thus selected for during tumor evolution? Several lines of evidence suggest that interpretation of the *Nlrp1*^129S^ variant as a cancer driver requires further functional validation: First, the pre-existing *Nlrp1*^129S^ variant does not arise through somatic mutation (it is already present in the germline of 129S-related mouse strains). LOH at the locus is explained by the tight genetic linkage of *Nlrp1*^129S^ to *Trp53*^ENG-129S^. Second, in our own cohorts of several hundred *Kras*^G12D^-driven mouse pancreatic cancers, there are no *Nlrp1* locus deletions without associated LOH of mutant *Trp53*^ENG^. Third, large transposon-based pancreatic cancer gene discovery screens performed on a mixed 129S;C57BL/6J genetic background did not find common insertions in the wild-type, hemizygous *Nlrp1*^C57BL/6J^ allele^9–11^. Fourth, in human pancreatic cancer, isolated, deep *NLRP1* deletions that spare *TP53*, have been only reported in 1/109 cases in one study (PDA_078 in UTSW cohort^12^; cbioportal.org). In fact, re-analysis of raw-sequencing data with manual inspection of the *NLRP1* locus in PDA_078 did not confirm the presence of an isolated *NLRP1* deletion in our hands.

Beyond the *Nlrp1* locus, our findings highlight an important—and so far underappreciated—confounder of mouse cancer genome analysis. The basis of this confounder is the widespread use of inbred mice with extensive inter-strain haplotype variation^5^. Typical experimental cohorts are derived from few generations of crosses involving few different inbred strains, with inheritance of large strain-specific haplotype blocks. Thus, when genetically linked to a cancer gene undergoing LOH, any strain-specific deletion/insertion variant will appear as a somatically acquired CNV in related tumors. Importantly, this CNV will be hugely recurrent in the cohort. Backcrossing of mice/alleles to a single genetic background can substantially reduce the amount of strain-specific germline haplotypes. However, this confounder can persist in direct genomic proximity to engineered alleles (which are bred/genotyped for) even after extensive backcrossing (Fig. 2c).

The general relevance of the considerations raised in our commentary is evident at many strain-specific loci linked to LOH of a cancer driver. The driver can be an engineered allele (like *Trp53*^ENG^), but also a somatically acquired cancer gene alteration. For example, in mouse pancreatic cancer cohorts we observed *Skint* locus deletions with equivalent characteristics to *Nlrp1* alterations: (1) identical genomic deletion coordinates in unrelated tumors, (2) corresponding strain-specific locus variation in the germline, and (3) genetic linkage to a cancer gene (*Cdkn2a*) undergoing LOH in the tumor. As for *Nlrp1*, the *Skint* locus deletion is present in the germline of 129S-related mouse strains but not in C57BL/6J (Fig. 2d, e).

In conclusion, our observations highlight the importance of considering sequence diversity of inbred mouse strains when analyzing cancer genomes in typical experimental settings/cohorts. With sequencing costs dropping, genomic analyses in mouse models of human cancer are increasing at a rapid pace. So far, strain-specific germline variants have obtained little attention in mouse cancer genome sequencing studies. Their potential interpretation as somatically acquired cancer drivers is a common problem, reinforcing the need to raise awareness of this confounder.

## Methods

### Datasets and data analyses

Data and conclusions of this commentary are based on the systematic genetic analysis of our own cohort of over 1000 mouse cancers derived from a variety of distinct mouse models covering different cancer entities. This large cohort of mouse cancers comprise primary cell cultures as well as tissues and was characterized by a series of methods, including array comparative genomic hybridization (aCGH), whole-exome sequencing (WES), long-read sequencing and/or quantitative insertion-site sequencing (QiSeq). Animal experiments, primary mouse pancreatic cancer culture preparation, and maintenance, gDNA isolation were performed as described in detail before^13,14^. Genome-wide identification of transposon integration sites in transposon-based mouse models was assessed by using QiSeq and custom bioinformatic analyses as described previously^11,15^. Animal studies were ethically approved by the Institutional Animal Care and Use Committees (IACUC) of Technische Universität München, Regierung von Oberbayern and the UK Home Office.

### Whole-exome sequencing

Raw WES data of mouse pancreatic cancers from our cohorts and from Niknafs et al.^1^ were analyzed by using a workflow adapted to the analysis of mouse cancer sequencing data which we described elsewhere in detail^14^ (source code: https://github.com/roland-rad-lab/MoCaSeq). In brief, reads were trimmed using Trimmomatic 0.38. BWA-MEM 0.7.17 was used to align reads to the mouse reference genome GRCm38.p6 (with alternate contigs). Picard 2.20.0 and GATK 4.1.0.0 were used for postprocessing (CleanSam, MarkDuplicates, BaseRecalibrator). For LOH analyses from WES data, germline SNP calling was performed with Mutect2 which removes the vast majority of sequencing artifacts. The high number of pseudogenes and segmental duplications in the mouse genome (as compared to the human genome) increases the chance of read mis-mapping. To avoid ambiguous SNP positions resulting from mis-mapping, only reads with a mapping quality of 60 were included in LOH analyses^14^. For CNV detection in mouse and human pancreatic cancers, we used CopywriteR 2.6.1.2^16^ which is based on the analysis of “off-target” reads. “Off-targets” (such as intronic reads), which represent ~20% of all reads in typical WES data sets (due to incomplete removal during standard library preparation), are not affected by variation in capture efficiencies. CopywriteR outperforms algorithms based on the analysis of “on-target” reads (exonic-read based algorithms) for CNV calling from human and mouse WES data^14,16^.

### Array comparative genomic hybridization

aCGH data from Niknafs et al.^1^, Maser et al.^2^, Foijer et al.^3^, Foijer et al.^4^, Cutler et al.^6^, Mueller et al.^13^ and our own KPC cohort was analyzed using Agilent Genomic Workbench software version 7.0.4.0. Importantly, the identical aCGH array (Agilent, SurePrint G3 Mouse CGH 240 K) was used in all studies, allowing for the direct comparison of strain-specific germline variation at *Nlrp1* and *Skint* loci across all mouse cancer cohorts/genomes.

### Long-read nanopore sequencing

Long-read sequencing libraries were prepared using the Oxford Nanopore LSK109 kit (ONT, Oxford, UK). A total of 400 ng of library was loaded on a promethION flowcell and run for 72 h on a promethION beta sequencer (ONT, Oxford, UK). Base-calling was performed on the promethION compute unit’s GPU using guppy 3.2.8 basecaller. The resulting FASTQ file was mapped to the reference genome GRCm38.p6 using Minimap2 (option *map-ont)*. Read coverage was extracted using bam-read count (*minimum mapping quality 60*, *minimum base quality 5*).

### Reporting summary

Further information on research design is available in the Nature Research Reporting Summary linked to this article.

## Supplementary information

Reporting Summary

## Data Availability

NGS and aCGH data from Niknafs et al.^1^ is available from the National Center for Biotechnology Information Sequence Read Archive (NCBI SRA) using study accession PRJNA546566 and from the Gene Expression Omnibus (GEO) database using the accession GSE132235. WES and aCGH data of our KC cohort, described by Mueller et al.^13^ is available from the European Nucleotide Archive using study accession PRJEB23787 and from the GEO database using accession GSE107458. Nanopore sequencing, WES and aCGH data of our KPC cohort is available from the European Nucleotide Archive using study accession PRJEB39427, PRJEB39429 and from the GEO database using accession GSE154537, respectively. aCGH data from Maser et al.^2^, Foijer et al.^3^, Foijer et al.^4^, and Cutler et al.^6^ is available from the GEO database using study accession GSE7615, GSE57334, GSE63686, and GSE9186, respectively. Human pancreatic cancer WES data of Witkiewicz et al.^12^ is available from NCBI SRA using study accession PRJNA278883.

## Code availability

The source code for WES analyses pipelines is available at https://github.com/roland-rad-lab/MoCaSeq.
